# The Effect of Decision Fatigue on Food Choices: A Narrative Review

**DOI:** 10.3390/nu17243901

**Published:** 2025-12-13

**Authors:** Natasha Brasington, Emma L. Beckett, Penta Pristijono, Taiwo O. Akanbi

**Affiliations:** 1 School of Environmental and Life Sciences, The University of Newcastle, Ourimbah, NSW 2258, Australia; penta.pristijono@newcastle.edu.au (P.P.); taiwo.akanbi@newcaslte.edu.au (T.O.A.); 2 Nutrition and Food Science, Australian Catholic University, 40 Edward Street, Sydney, NSW 2060, Australia

**Keywords:** decision fatigue, food choices, ego depletion, convenience foods

## Abstract

**Background:** Decision fatigue has been studied in medical settings but is rarely explored in the context of food choice. Decision fatigue can lead to depleted mental energy, exhaustion, poorer decision-making abilities, reduced willpower, increased risk aversion, and impaired prioritisation. Food choices are frequent decision-making situations that may be influenced by decision fatigue, potentially leading to impulsive and less health-conscious food selections. **Objective:** This review consolidates current knowledge on food choice and its complexity, decision fatigue, and ego depletion, and considers how these may interact. The scales used to measure decision fatigue are also reviewed. **Methods:** Literature was identified through searches in PubMed and Google Scholar databases using combinations of keywords such as “decision fatigue,” “ego depletion,” “food choice,” and “dietary behaviour.” Relevant studies were screened based on English-language publications and relevance to food choice. **Results:** Existing studies highlight associations between decision fatigue and poorer decision quality, but few have directly examined links to food choice. Evidence suggests potential mechanisms, though findings remain largely speculative. **Conclusions:** There is currently a paucity of evidence specifically connecting decision fatigue with food choices. Potential solutions to reduce this burden, such as the use of convenience foods as a nudge toward healthier decisions, warrant further exploration.

## 1. Introduction

Diet is a major modifiable determinant of health and disease. Consuming a balanced and nutrient-dense diet has been known to reduce the risk of chronic diseases and can promote long-term wellbeing [1]. Despite well-known dietary outcomes, everyday eating behaviours remain complex and difficult to change [2,3,4]. Individuals navigate hundreds of food-related decisions each day, which include what, when, and how to eat, which are often in environments that compete for attention and contain numerous cues that then influence behaviour [5,6,7,8]. This continual decision-making creates a substantial cognitive load on individuals, helping to explain why knowledge alone rarely leads to behaviour change. Food choices can be influenced by a broad range of factors, including individual, social, environmental, and macro-level contexts [5,6,7].

Importantly, many of these decisions occur when an individual is distracted or in stressful situations where cognitive capacity is already strained, increasing the likelihood of “mindless eating” and suboptimal choices [1]. The following topic aligns with theories of ego depletion, which suggests that self-regulation is a limited resource that can become reduced following repeated or effortful decision-making [9]. When self-regulatory capacity is diminished, individuals are more prone to selecting automatic, impulsive responses and less able to engage in effortful, reflective choices, which is an effect particularly relevant to food selection.

Research indicates that people who are susceptible to cognitive fatigue are not evenly distributed across populations. Fatigue effects tend to be stronger among individuals experiencing chronic stress, socioeconomic disadvantage, and high daily decision loads, such as shift workers, caregivers, and low-income households managing constrained resources [10,11].

Younger adults also show greater vulnerability to self-regulatory depletion, particularly in contexts involving consumer behaviour and impulse-driven food choices [12], and these patterns suggest that both age and social context shape how strongly depletion influences decision-making and dietary behaviour.

Ego depletion and decision fatigue processes have been studied in medical and judicial contexts [13,14,15,16,17], but are underexplored within the nutrition field, despite the rising complexity of modern food environments [8,18]. Supermarkets now have an endless list of products, promotions, and nudges that can increase cognitive load and influence behavioural defaults. But it remains unclear how cognitive strain links with these environments to shape real-world food choices, representing a meaningful gap in existing research.

This narrative review examines the relationship between decision fatigue, ego depletion, and food choice. The review aims to synthesise current evidence, identify conceptual and methodological gaps, and highlight the need for stronger research linking cognitive load to everyday eating behaviour. Defining the mechanisms by which decision fatigue may influence dietary patterns, this review seeks to support future work that can improve nutrition interventions and ultimately support healthier food choices. This review offers a novel contribution by integrating decision fatigue theory with established food-choice frameworks and highlighting how cognitive load may influence eating behaviour. To date, no published review has examined convenience cooking products and decision fatigue, despite their increasing role in modern dietary patterns. By identifying this conceptual intersection and mapping key gaps in empirical evidence, this review provides a foundation for future research exploring how cognitive strain and convenience-based food choices interact to influence dietary behaviour.

## 2. Methods

### 2.1. Review Approach and Rationale

The use of a narrative review was selected as the literature on decision fatigue and food choice is multifaceted, unsystematic, and is found in multiple disciplines. These outcomes make quantitative synthesis unfitting. Therefore, a narrative approach allows the inclusion of theoretical, experimental, and applied evidence to identify conceptual links, highlight gaps, and propose directions for future research.

### 2.2. Search Strategy

The literature search was conducted from April 2025 to November 2025 using PubMed and Google Scholar. The search included free-text terms related to decision fatigue and food choice. Core keyword combinations included were as follows: “decision fatigue”, “ego depletion”, “cognitive load”, “self-control depletion”, “food choice”, “dietary behaviour”, and “convenience foods”. Additional articles were included by screening the reference lists of key papers. No systematic review software or data analysis software was used in this narrative review. EndNote 20 (Clarivate, Philadelphia, PA, USA) was used solely for reference management.

### 2.3. Eligibility Criteria

Studies were screened according to predefined inclusion and exclusion criteria (Table 1).

### 2.4. Screening Process

**Figure 1:**
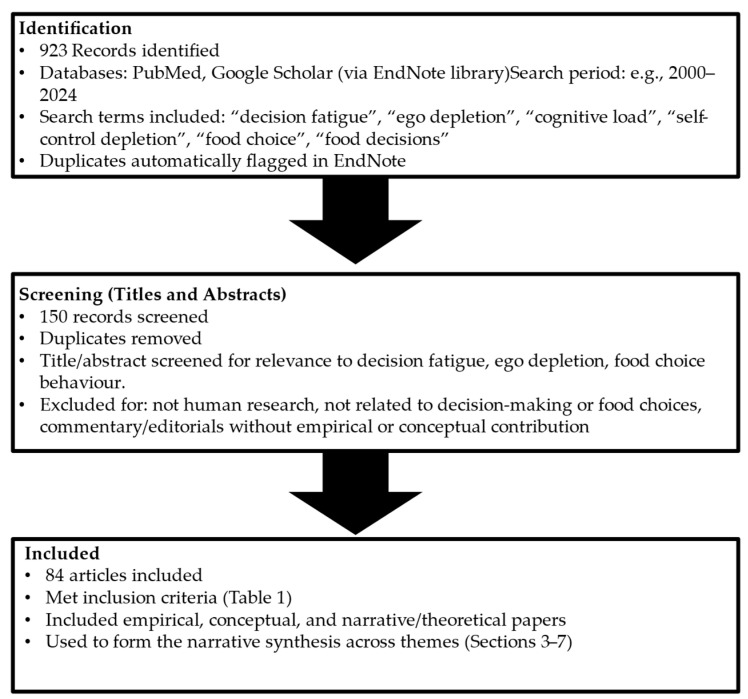
Flow diagram of narrative review process.

A total of 923 records were identified through database searches and reference list screening. Titles and abstracts were screened for importance to decision fatigue, ego depletion, or food choice, resulting in 150 records kept for full-text assessment. Duplicates and irrelevant articles were removed. Full-text screening was conducted by the first author using the predefined eligibility criteria (Table 1). With uncertainties, decisions were discussed with a senior academic colleague to check consistency and transparency in study selection. Following full-text assessment, 84 articles had met the inclusion criteria and were included in the final synthesis (Figure 1).

### 2.5. Quality Appraisal

Formal risk-of-bias tools were not fully applicable because of the diverse designs; instead, a brief structured appraisal was conducted. Each study was assessed for its clarity of aims, appropriateness of study design, adequacy of sample size, transparency of methods, validity of measures, and relevance to everyday food choice contexts.

### 2.6. Synthesis of Evidence

Studies were grouped into four analytic categories, which included determinants of food choice, decision fatigue, and ego depletion mechanisms, measurement instruments for fatigue, and how decision fatigue may influence food choice behaviour.

Key characteristics, methods, exposure types, and outcomes across included studies are summarised in tabular format. The synthesis emphasised conceptual integration, identification of gaps, and implications for understanding food-related decision-making under cognitive load.

## 3. Results

### 3.1. Food Choices

Food choices are multifaceted and complex phenomena [19]. Why and how individuals select foods has been extensively studied, with Furst et al. identifying five major categories influencing food choice, which include cultural factors, personal factors, resources, social factors, and present contexts [20]. Although these factors appear simple, food decisions can occur rapidly and repeatedly throughout the day, placing substantial cognitive demands on individuals. This complexity highlights why nutritional knowledge alone can rarely lead to sustained behaviour change and may explain why mental fatigue may shape dietary patterns, particularly when decisions are made under distraction or stress.

Research in nutrition, sensory science, marketing, sociology, agricultural economics, and behavioural economics provides many outlooks on food choice [18]. However, much of this work tends to be descriptive and often does not account for how individuals make decisions when cognitive resources are restricted [19]. Existing food-choice frameworks tend to assume that people can consciously evaluate options, an assumption that becomes problematic in the context of cognitive load or decision fatigue. Table 2 summarises foundational and contemporary evidence across individual, social, environmental, and structural domains, illustrating both the breadth of research and the gaps related to cognitive strain.

The socio-ecological model (SEM) is widely used to conceptualise influences on eating behaviour across multiple levels [24]. It effectively captures environmental, interpersonal, organisational, and policy determinants of diet [25,26,27,28], but it does not explicitly address how cognitive processes determine which level becomes most influential when individuals face heavy decision load, time pressure, or depletion. As a result, SEM describes the scope of influences but offers limited insight into how cognitive fatigue may shift behaviour toward defaults, habits, or convenience options.

Townsend and Foster’s application of SEM to school food environments demonstrated that interpersonal influences and school policies strongly shape students’ lunch choices [29]. However, the framework did not consider whether these choices varied as cognitive burden increased throughout the day. This omission reflects a broader limitation of existing food-choice models: they identify determinants but rarely consider the mental effort required to navigate them.

**Figure 2:**
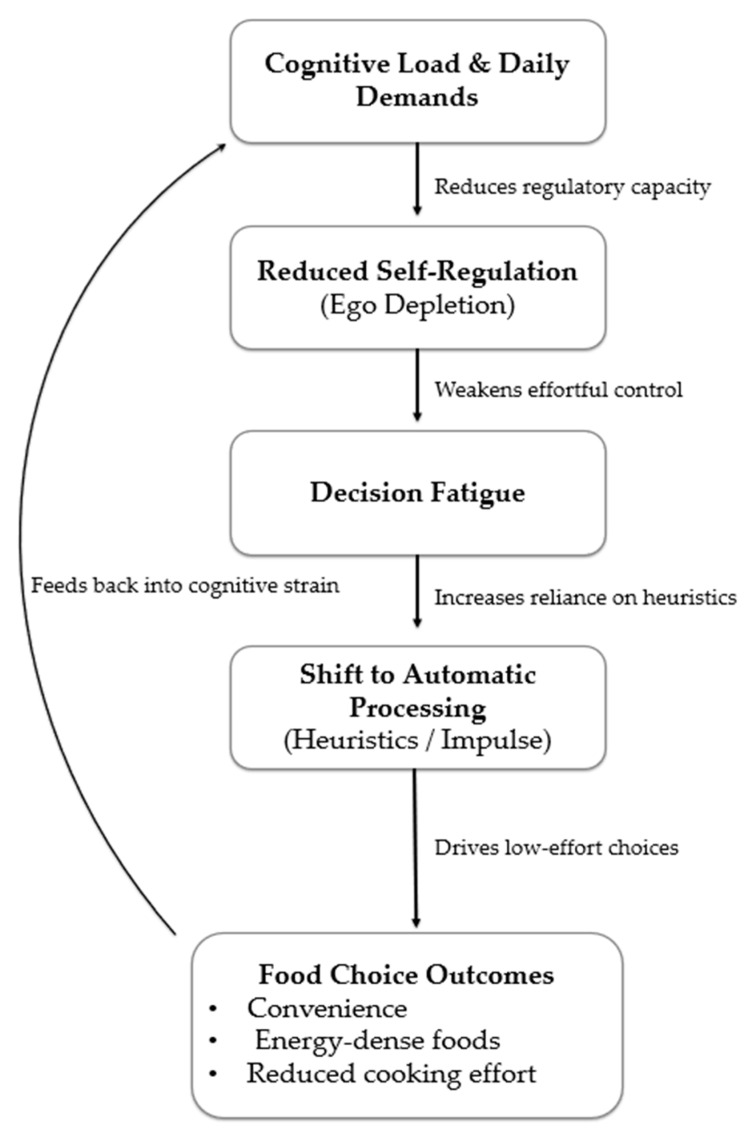
Conceptual model showing how cognitive load reduces self-regulation, leading to decision fatigue, a shift to automatic processing, and lower-effort food choices.

Similarly, the Determinants of Nutrition and Eating (DONE) framework provides a comprehensive classification of individual, social, environmental, and policy-level influences [30,31]. While valuable for mapping determinants, it treats these as relatively stable categories and does not specify how individuals prioritise them when cognitively depleted or required to make rapid decisions. Such scenarios, including distraction, time pressure, or sequential decision load, are precisely the contexts in which decision fatigue is most likely to influence food choices. To illustrate how cognitive load may interact with these determinants, Figure 2 presents a conceptual model linking high decision load and reduced self-regulation to shifts in food-choice behaviour.

These frameworks underscore the complexity of food choice but also reveal a significant conceptual gap. They describe what influences food choice but not how cognitive strain then alters the weighting, sequencing, or accessibility of those influences in real-world settings. Understanding this interaction is essential for interpreting why individuals may rely more heavily on convenience foods or heuristic-driven choices when they are mentally fatigued. Future research should include decision fatigue processes into existing food-choice frameworks to better explain how cognitive load shapes everyday eating behaviour.

Food choice has evolved in response to shifting food systems, changing availability, and broader social and cultural transitions [32,33,34]. These changes influence not only what foods are consumed but also how individuals then navigate increasingly complex food environments. Many food decisions are made rapidly and with minimal deliberation, and even subtle mood shifts can alter preferences [35]. As consumers look for factors such as taste, health, convenience, cost, ethics, and environmental considerations, food choice becomes a multifactorial and cognitively demanding process [36,37]. Decisions may also require planning across meals, linking past, present, and future eating episodes, adding further cognitive burden [38]. Brunner et al. [22] further showed that modern consumers increasingly rely on convenience foods as a way to reduce time pressure and cognitive effort, illustrating how environmental complexity and mental load shape everyday eating patterns.

Food-related decisions can occur automatically, habitually, or deliberately with effortful reasoning. Many everyday food choices, such as selecting a quick snack, can be driven by fast, cue-responsive, and largely unconscious processes. However, more proposed choices require reflective thinking, such as evaluating nutritional value, cost, or long-term health considerations [39,40]. These dual pathways tend not to be equally accessible at all times. Subtle nudging and priming cues can shift food choices with minimal cognitive effort. Wilson et al. showed that simple environmental prompts reliably influence purchasing, particularly when individuals rely on automatic decision processes.

This contrast is important for understanding the potential influence of decision fatigue. As cognitive resources become depleted throughout the day, individuals are more likely to rely on automatic, effortless strategies rather than reflective decision-making. These conditions lead to convenient, energy-dense, or immediately rewarding foods, even when such options conflict with their long-term goals. Evidence from ego depletion research shows that reduced self-control is associated with more impulsive behaviour across multiple domains [12,41,42,43,44,45,46,47]. However, direct studies linking decision fatigue to real-world food choices are limited, indicating a key gap for future research.

### 3.2. Decision Fatigue and Ego Depletion

Decision fatigue, ego depletion, and general fatigue are related but conceptually distinct constructs. Fatigue is known as physical, emotional, or cognitive tiredness and is typically assessed using validated fatigue scales. Decision fatigue, sometimes known as “choice overload,” is the deterioration in judgement quality that occurs after repeated or effortful decision-making [48,49,50,51]. Ego depletion, derived from Baumeister’s Strength Model of Self-Control [9], looks at a temporary reduction in self-regulation following exertion. Decision fatigue can, therefore, be understood as a context-specific aspect of ego depletion, particularly relevant in areas that require continuous, sequential decision-making, such as food selection; see Table 3 for an overview of foundational and contemporary studies examining decision fatigue and ego depletion across various behavioural domains. [52].

Decision-making relies on limited cognitive resources, and when these resources are depleted, people shift towards faster, automatic, and low-effort responses. Baumeister’s early laboratory work established that self-control operates as a limited resource, with performance diminishing after sequential self-regulatory tasks [9].

Subsequent work expanded this evidence: Vohs and Faber showed that depleted consumers engage in greater impulse buying, Schmeichel et al. reported impairments in higher-order reasoning, and Ainsworth et al. demonstrated that depletion reduces trust in economic exchanges. Collectively, these studies indicate that depletion undermines both cognitive clarity and behavioural regulation.

Real-world decision settings illustrate these effects. In judicial contexts, Danziger et al. found that judges increasingly defaulted to denying parole as decision load accumulated across the day is a classic pattern of decision fatigue. Another study found that harsher bail decisions were made later in case sequences. These findings show that depletion reduces deliberation and increases reliance on risk-averse options [17].

Food-related behaviours follow the same behavioural logic. Vohs and Heatherton demonstrated that depleted restrained eaters consumed significantly more ice cream, illustrating how ego depletion heightens susceptibility to palatable, high-calorie foods. Together, these studies reinforce that depletion shifts behaviour toward immediacy, reward seeking, and minimal cognitive effort.

Decision fatigue is particularly relevant for food choice because eating involves frequent, sequential decisions on what to eat, when, how much, whether to cook, and how to prepare food, with individuals making dozens to hundreds of these choices each day [53]. When cognitive resources are intact, individuals can evaluate options in line with long-term health goals. However, as resources become depleted, behaviour becomes increasingly shaped by convenience, habit, environmental cues, and automatic responses [54].

Pignatiello et al. emphasise that although the term “decision fatigue” was not originally used by Baumeister, his model of limited regulatory resources directly predicts this phenomenon. Once regulatory resources are diminished, performance declines across domains, including reasoning, emotional regulation, impulse control, and reflective decision-making. These actions define why individuals experience decision fatigue and why they tend to go for convenient or energy-dense foods even when motivated to eat healthily.

Therefore, general fatigue can show multiple dimensions of tiredness, decision fatigue then highlights the specific cognitive burden of repeated decisions, and ego depletion provides the theoretical foundation for understanding how these burdens alter food-related behaviour.

### 3.3. Measures and Scales of Decision Fatigue

Many fatigue scales have been developed across clinical and research settings, targeting different dimensions of tiredness, including physical, cognitive, emotional, and psychosocial fatigue. However, because decision fatigue is a relatively recent construct, there remains a notable lack of instruments that are specifically designed to measure it.

Although most tools used in nutrition and behavioural research rely on explicit self-report scales, decision fatigue-related constructs have also been assessed using implicit or behavioural tasks in psychological research. These include response-time paradigms, Stroop interference tasks, Go/No-Go inhibition tasks, and working-memory depletion tasks, which indirectly capture self-regulatory decline and cognitive load [44,45]. While not designed as formal decision fatigue measures, these tasks provide valuable insight into the cognitive mechanisms underpinning depletion and represent potential methodological avenues for future work in food-choice settings.

Most existing tools assess general fatigue rather than the cognitive burden associated with repeated decision-making. The most widely used instrument in this area is the 10-item Decision Fatigue Scale (DFS) [55], yet even this tool was developed for a very specific clinical context. Table 4 summarises the commonly used fatigue and decision fatigue scales, outlining their purpose, target populations, structure, and key strengths and limitations.

General fatigue scales capture broad aspects of tiredness and do not directly assess reduced decision-making capacity or self-regulatory decline. While these measures can detect cognitive fatigue, their outcomes differ from those of decision fatigue tools, which are designed to assess altered cognitive processing, difficulty evaluating information, and increased impulsive responding. Nonetheless, general fatigue scales offer a starting point for understanding related constructs and may inform the development of more targeted decision fatigue measures.

The Fatigue Assessment Scale (FAS) is a 10-item unidimensional tool that uses a 5-point Likert scale to assess both physical and mental fatigue [56]. Initially developed for use in large working populations and later validated among individuals with sarcoidosis, the FAS is quick to administer and demonstrates satisfactory reliability. However, it is not intended to assess decision-related cognitive decline.

The Multidimensional Fatigue Inventory (MFI-20) includes 20 items across five dimensions: general fatigue, physical fatigue, reduced motivation, reduced activity, and mental fatigue [57]. Although widely used across clinical and non-clinical populations, evidence indicates structural limitations. Wintermann et al. [60] found poor reliability in certain populations, especially for the reduced motivation dimension, and Hinz et al. [61] highlighted an unclear factor structure, suggesting item reduction may strengthen validity.

The Fatigue Severity Scale (FSS) is a nine-item unidimensional scale developed for populations with neurological conditions such as multiple sclerosis and lupus. It focuses on how fatigue interferes with daily functioning and uses a 7-point Likert response format. Although widely used and time-efficient, it does not capture the cognitive mechanisms associated with decision fatigue.

The Pittsburgh Fatigability Scale (PFS) is a 10-item measure that differentiates between physical and mental fatigability, with scores reflecting fatigue experienced after specific activities. This activity-based approach offers insight into mental exertion but does not explicitly evaluate decision-related cognitive impairment.

Overall, most validated fatigue scales were designed for clinical populations and do not capture the cognitive mechanisms underlying decision fatigue or ego depletion. Their broad scope means individuals with similar overall scores may experience distinct cognitive challenges, limiting their usefulness in understanding decision-making contexts. There is a clear need for measures that assess the specific cognitive, emotional, and behavioural components of decision fatigue in everyday settings, including food-related decisions.

The most widely recognised decision fatigue instrument is the 10-item Decision Fatigue Scale (DFS), developed by Hickman et al. [55] for surrogate decision-makers of critically ill patients. The DFS assesses emotional distress, mental exhaustion, impaired information processing, increased decision effort, and impulsive decision-making. Items are rated on a 4-point Likert scale, and total scores range from 0 to 30, with higher scores indicating greater perceived decision fatigue. Although designed for clinical decision-making, the DFS offers a relevant foundation for measuring decision fatigue in other contexts, including everyday food choices and the use of convenience products. Its readability and simplicity suggest potential applicability beyond healthcare settings, though further validation is required.

### 3.4. How Can Decision Fatigue Influence Food Choices?

Decision fatigue can lead to reliance on fast, automatic, and impulsive decision-making processes, which can cause individuals to prioritise short-term rewards and default options that require minimal cognitive effort [62,63]. Under these conditions, people are more susceptible to external cues, advertisements, and convenience-driven prompts [62,64]. In food environments, this often manifests as selecting readily available items, for example, snacks placed at checkouts or end-of-aisle displays, which cater to immediate desire rather than long-term intentions [65,66]. Table 5 provides a summary of key studies examining the relationship between cognitive load, decision fatigue, and ego depletion and suboptimal food choices and impulsive dietary behaviours.

Experimental work by Wang et al. similarly demonstrates that depleted individuals show a clear shift toward indulgent and energy-dense foods, reinforcing the tendency toward short-term reward seeking under cognitive load. Across daily life, individuals encounter an abundance of food and drink options in homes, workplaces, convenience stores, petrol stations, and supermarkets. While broad availability can support autonomy and satisfaction, it also increases exposure to cues that promote immediate gratification. These cues interact with mood, distraction, sensory signals, and social influences, contributing to patterns of choice that reflect cognitive load more than physiological need [23]. As a result, decision fatigue can amplify tendencies toward overeating, preference for energy-dense foods, and reduced capacity to make reflective, health-oriented decisions. Building on this, Salmon et al. found that ego-depleted participants were significantly more likely to purchase unhealthy snacks, highlighting how decision fatigue can shape purchasing behaviour in real-world food environments.

The concept of willpower is closely aligned with decision fatigue. As cognitive resources become depleted, self-control diminishes, increasing vulnerability to temptations and reducing the ability to resist convenient, hedonic foods. Nudging strategies aim to shift these tendencies by subtly restructuring environments to guide behaviour without restricting options [74]. In food settings, nudges such as changing product order, altering shelf placement, or increasing the visibility of healthier items have demonstrated measurable impacts on dietary choices [71,72].

Field evidence reinforces these patterns. The WRAPPED supermarket trial showed that placing fruit and vegetables closer to store entrances increased purchasing behaviour [75]. Gillebaart et al. demonstrated that easy-to-grab vegetable snack packs at checkouts increased vegetable purchases, while Bauer et al. found that sequential cafeteria interventions modestly improved healthier selections. The Click and Crunch school canteen trial further highlighted how multi-component choice architecture strategies could improve students’ lunch purchases in the short term [76].

Food aversion illustrates another mechanism through which cognitive and emotional responses shape eating behaviour. Negative experiences with food can create strong, persistent avoidance patterns after only a single episode [77,78,79]. Although less common than general food dislikes, aversions can strongly influence habitual eating. Habits themselves are powerful drivers of food choices and are shaped by childhood environments, parental influence, peer norms, and personal experiences [80]. Over time, habits may shift from deliberate decisions to automatic responses triggered by environmental cues.

Convenience cooking products provide an important point of view through which to understand how decision fatigue may shape everyday food choices. Convenience foods have long been associated with time pressure, reduced cognitive effort, and the desire to minimise meal preparation demands. Brunner et al. [22] identified convenience as a major driver of modern food purchasing, demonstrating that individuals often choose convenience products to reduce the cognitive and practical burdens of planning and cooking. This tendency aligns with broader evidence showing that consumers frequently rely on effort-reducing strategies when cognitive load is high, leading them to choose foods that simplify decision-making and preparation [21].

Recent empirical work reinforces these patterns. Brasington et al. [81,82] found that frequent users of convenience cooking products tend to report lower cooking confidence and creativity, and have reduced vegetable intake, suggesting that reliance on such products may reflect or reinforce low cognitive bandwidth for meal preparation. Moreover, health professionals surveyed in Brasington et al. [73] identified convenience products as a practical solution during periods of high decision demand, explicitly linking cognitive load to reliance on these products. Together, this evidence positions convenience cooking products as a plausible behavioural response to decision fatigue, rather than a speculative assumption.

Several mechanisms support this interpretation. Convenience products reduce the number of decisions required during meal preparation (e.g., selecting ingredients, choosing cooking methods, and estimating quantities), which is consistent with decision fatigue theory predicting a shift toward low-effort, high-structure options under cognitive strain [9,12]. These products often include step-by-step instructions and simplified ingredient lists, further reducing decision complexity and reliance on self-regulatory resources. This aligns with broader findings in food-choice research showing that individuals gravitate toward default or low-effort options when cognitively depleted [23,67,68,69].

Although direct empirical tests of decision fatigue and convenience product use remain limited, the converging evidence from food-choice behaviour, cognitive-load research, and consumer preferences provides a theoretical and empirical foundation for this proposed connection. Strengthening the theoretical alignment in this way supports the argument that convenience cooking products may function as a self-regulatory tool during periods of high cognitive load, helping individuals manage everyday decision demands related to food.

### 3.5. Discussion

A critical evaluation of the included literature shows considerable methodological limitations and inconsistencies, which indicate that current evidence on decision fatigue and food choice remains inadequate for strong conclusions. Although theoretical support for ego depletion and decision fatigue is strong, much of the empirical evidence is derived from laboratory-based studies using artificial tasks, student samples, and short-term manipulations. These designs limit external validity and reduce confidence in how well findings translate to real-world food environments.

Across studies, measurement inconsistency is a major challenge. Fatigue and depletion are implemented using diverse instruments, which include depletion tasks, self-report scales, time-of-day proxies, cognitive-load manipulations, and behavioural outcomes that lack validation for use in everyday food-choice contexts. Decision fatigue has only been applied in medical or critical-care settings. This mix of measurements makes comparison across studies difficult and contributes to inconsistent findings.

In addition, very few studies investigate decision fatigue directly in relation to food decisions. The most relevant evidence is from ego depletion research or from consumer-behaviour studies focused on impulsive or convenient choices. These findings provide limited hands-on support for how decision fatigue influences everyday eating patterns, particularly in complex food environments like supermarkets or home settings.

The existing literature tends to be characterised by narrow populations and limited diversity. Many studies rely on young, educated, Western samples, restricting generalisability. Real-world decision fatigue has been studied more extensively in judicial and clinical contexts than in nutrition, leaving gaps. Convenience cooking products, which are highly relevant to modern dietary behaviour, have not been tested in relation to cognitive load, with only recent conceptual evidence emerging.

Finally, few studies assess long-term effects and how repeated daily decisions accumulate to influence food choices over time. It remains unclear whether decision fatigue consistently predicts poorer dietary outcomes or interacts with environmental, emotional, or habitual factors. Overall, the current evidence highlights meaningful theoretical pathways but lacks methodological and consistent measurement and real-world applications. These gaps limit firm conclusions and underscore the need for systematic, multidisciplinary research that tests decision fatigue in ecological food environments and evaluates interventions designed to reduce cognitive load during food choice.

## 4. Strengths and Limitations

This narrative review provides a comprehensive synthesis of theoretical, experimental, and applied evidence linking decision fatigue, ego depletion, and food choice. A key strength of this review is its multidisciplinary perspective, drawing from psychology, nutrition, behavioural economics, and consumer research to integrate concepts that are not often examined together. With the integration of these foundational theories and the addition of the emerging evidence from food environments and convenience cooking products, the review identifies novel conceptual pathways and highlights underexplored sections in the literature. The search strategy allowed for the identification of studies across diverse settings and contributed to a more holistic understanding of cognitive load shaping everyday eating behaviours.

There are several limitations that must be acknowledged. First, as this is a narrative literature review, the synthesis is interpretive rather than systematic, and the absence of formal risk-of-bias assessment tools limits the ability to evaluate methodological quality across studies. There were limited measurement consistencies across studies, ranging from general fatigue scales to varied depletion models, which limit comparability and synthesis. Also, a few studies directly examine decision fatigue and food choices, which has led to a more theoretical estimation rather than direct factual evidence. The literature on convenience cooking products is also limited, and the current findings tend to be more conceptual. This review may not have captured all relevant evidence due to discipline-specific terminology.

Together, these strengths and limitations highlight both the conceptual value and the empirical gaps in the existing research. Continued interdisciplinary work, particularly in real-world food environments, is needed to substantiate the mechanisms proposed in this review.

## 5. Conclusions

Food choice is extremely complex. It is shaped by interacting with individual, social, environmental, and policy-level influences. Although decision fatigue is well-established within psychology and is supported by robust evidence from medicine, economics, and judicial research, it remains understudied in the context of everyday eating. Existing findings suggest that depleted self-control increases reliance on automatic, impulsive responses that may translate into less healthy dietary behaviours. However, direct evidence linking decision fatigue to real-world food decisions is still limited.

This gap highlights the need for research that tests whether mechanisms observed in laboratory and clinical settings, such as ego depletion, cognitive load, and choice overload, operate similarly in food environments. Emerging strategies such as nudging interventions and the use of convenience cooking products show promising avenues for reducing the cognitive burden of food choice, but they are largely untested within the decision fatigue framework. These observations represent hypotheses that require empirical evaluation. Overall, decision fatigue offers a compelling conceptual outlook for understanding why individuals may struggle to consistently make health-promoting food choices in modern, high-load environments. This shows its role within everyday dietary behaviour and how it is crucial to design interventions that align with the realities of contemporary food settings and support sustainability in healthier eating patterns.

This review is the first to position convenience cooking products within a decision fatigue framework, highlighting a novel area for empirical investigation.

## Figures and Tables

**Table 1:** Summary of inclusion and exclusion criteria.

Criteria	Defined
Inclusion	Studies exploring decision fatigue, ego depletion, or choice overload; studies exploring food choice, dietary decisions, eating behaviour, systems linking cognitive load to food-related outcomes, and human research; and studies written in English.
Exclusion	Studies focused only on physical fatigue, clinical fatigue conditions, metabolic/biological outcomes, taste aversion, animal studies, or unrelated nutrition physiology and non-English publications.
Filters	Literature published within the past 20–25 years, and the inclusion of seminal theoretical papers when they are foundational to decision fatigue mechanisms.

**Table 2:** Highlights of research in food choice.

Reference	Focus/Exposure Type	Population/Context	Key Insight
Furst et al., 1996 [20]	Food choice process model	General population	Identifies food choice as a dynamic, multi-stage process shaped by personal, social, and contextual factors.
Sobal & Bisogni, 2009 [19]	Food decision construction	Adults	Shows how food choices are constructed through values, identity, and routines.
Symmank et al., 2017 [21]	Mapping predictors of food decisions	Multidisciplinary review	Summarises individual, situational, and environmental influences on food decisions relevant to cognitive load.
Brunner et al., 2010 [22]	Convenience food consumption drivers	Adults	Shows time pressure, convenience needs, and effort reduction strongly shape modern food choices.
Wilson et al., 2016 [23]	Salience/priming nudges	Retail environments	Simple environmental prompts influence purchasing with minimal cognitive effort.

**Table 3:** Highlights of research in decision fatigue and ego depletion.

Reference	Design/Exposure	Population	Outcome	Key Insight
Baumeister et al., 1998 [9]	Sequential self-control tasks	Students	Self-control performance	Evidence that self-control operates as a limited resource.
Vohs & Faber, 2007 [12]	Ego depletion and consumer spending	Adult consumers	Impulse buying	Depleted individuals spend more and resist temptations less.
Schmeichel et al., 2003 [44]	Cognitive self-control task	Students	Logical reasoning	Depletion impairs higher-order cognition.
Vohs & Heatherton, 2000 [45]	Emotion regulation and eating	Restrained eaters	Ice cream consumption	Depleted individuals overeat, especially restrained eaters.
Pignatiello et al., 2020 [52]	Conceptual analysis	Review	Framework definition	Clarifies components of decision fatigue (cognitive, emotional, and behavioural).
Danziger et al., 2011 [13]	Time-of-day decision fatigue	Judges	Parole decisions	Decision quality drops over time, and default options increase.
Torres & Williams, 2022 [17]	Case-sequence fatigue	Judges	Bail decisions	Later decisions are more conservative/harsh.
Ainsworth et al., 2014 [42]	Ego depletion and trust	Adults	Economic trust	Depletion reduces trust and prosocial decision-making.

**Table 4:** Summary of fatigue and decision fatigue scales.

Scale	Purpose/Population	Dimensions	No. of Items	Response Format	Strengths	Limitations
Fatigue Assessment Scale (FAS) [56]	General fatigue in the working population: validated in sarcoidosis.	Unidimensional: physical and mental fatigue.	10	5-point Likert	Simple, quick, and reliable	Not decision fatigue specific
Multidimensional Fatigue Inventory (MFI) [57]	Severity of fatigue in various populations: cancer, military, and students.	5 dimensions: general, physical, cognitive, reduced motivation, and reduced activity.	20	5-point Likert	Broad scope, multidimensional	Structure questions, and less reliable in “motivation”
Fatigue Severity Scale (FSS) [58]	Fatigue has an impact on neurological disorders, such as multiple sclerosis and systemic lupus erythematosus.	Unidimensional.	9	7-point Likert	Widely used, simple	Disease-specific, not tailored to cognitive decision fatigue
Pittsburgh Fatigability Scale (PFS) [59]	Fatigability (physical and mental) across activities.	2 dimensions.	10	Activity ratings (0–50)	Distinguishes physical versus mental fatigue	Developed for older adults, not decision-specific
Decision Fatigue Scale (DFS) [55]	Decision fatigue in surrogate healthcare decision-makers.	Emotional distress, mental exhaustion, and impulsivity.	10	4-point Likert	Tailored to decision-making and good readability	Limited use outside medical settings

**Table 5:** Highlights of research in decision fatigue and food choice.

Reference	Exposure/Manipulation	Population	Food-Related Outcome	Key Insight
Vohs & Heatherton, 2000 [45]	Emotion regulation and depletion	Restrained eaters	Ice cream consumption	Depleted individuals consume more high-calorie foods.
Haynes et al., 2016 [67]	Ego depletion (lab task)	Adults	Unhealthy snack intake	Depletion increases unhealthy eating for those high in depletion sensitivity.
Wang et al., 2016 [68]	Depletion task	Adults	Unhealthy food preference	Depleted participants select more indulgent foods.
Salmon et al., 2014 [69]	Self-control depletion	Adults	Unhealthy snack purchases	Depletion predicts a higher likelihood of unhealthy purchasing.
Olsen et al., 2017 [70]	Time of day as proxy for DF	Adults in an online food choice task	Food choice behaviour	Later-in-day choices show patterns consistent with fatigue, which lead to default/unhealthy choices.
Musher-Eizenman et al., 2010 [71]	Food distance/accessibility	Children	Snack intake	Lower-effort options are chosen more, aligning with fatigue-driven decision simplification.
Maas et al., 2012 [72]	Accessibility manipulation	Adults	Snack consumption	Increased physical effort reduces consumption consistently with cognitive conservation.
Brasington et al., 2025 [73]	Convenience cooking products	Health professionals	Perceived impact of DF on food decisions	Professionals report DF as a driver of convenience food use; conceptual real-world relevance.

## Data Availability

Data sharing is not applicable to this article as no new data were created or analysed in this study.
